# Electronic states in GaAs-(Al,Ga)As eccentric quantum rings under nonresonant intense laser and magnetic fields

**DOI:** 10.1038/s41598-018-38114-0

**Published:** 2019-02-05

**Authors:** J. A. Vinasco, A. Radu, E. Niculescu, M. E. Mora-Ramos, E. Feddi, V. Tulupenko, R. L. Restrepo, E. Kasapoglu, A. L. Morales, C. A. Duque

**Affiliations:** 1 Grupo de Materia Condensada-UdeA, Instituto de Física, Facultad de Ciencias Exactas y Naturales, Universidad de Antioquia UdeA, Calle 70 No. 52-21, Medellín, Colombia; 2 Department of Physics, “Politehnica” University of Bucharest, 313 Splaiul Independenţei, Bucharest, RO-060042 Romania; 3 Centro de Investigación en Ciencias, Instituto de Investigación en Ciencias Básicas y Aplicadas, Universidad Autónoma del Estado de Morelos, Av. Universidad 1001, CP 62209 Cuernavaca, Morelos Mexico; 4 Laboratoire de la Matière Condensèe et Sciences Interdisciplinaires (LaMCScI) Group of Optoelectronic of Semiconductors and Nanomaterials ENSET, Mohammed V University in Rabat, Rabat, Morocco; 5 Donbass State Engineering Academy, Kramatorsk, 84313 Ukraine; 6 Universidad EIA, CP 055428 Envigado, Colombia; 7 Faculty of Science, Department of Physics, Cumhuriyet University, 58140 Sivas, Turkey

## Abstract

The features of the electron energy spectrum in eccentric two-dimensional GaAs-AlGaAs quantum rings of circular shape are theoretically investigated taking into account the effect of externally applied magnetic and intense laser fields. Analytical expressions for the laser-dressed confining potential in this kind of quantum ring geometry are reported for the first time. Finite element method is used to solve the resulting single-particle effective mass two-dimensional partial differential equation. It is shown that the allowed level spectrum is greatly influence by the external probe as well as by the breaking of geometric symmetry related to the changes in eccentricity. In presence of an intense laser field, the conduction band confining profile suffers strong modifications along the structure, with an additional contribution to symmetry breaking. These modifications of electronic quantum states reflect in the intraband optical absorption. Accordingly, the features of the intraband transitions are discussed in detail, revealing the significant influence of the magnetic field strength and laser field intensity and polarization, together with eccentricity, in the allowing of ground-to-excited states transitions and their corresponding intensities.

## Introduction

Semiconductor quantum rings (QRs) are nanoscopic structures which have emerged as promising systems for applications in physics and technology due to their particular electronic and optical properties. Being doubly-connected structures, their physical properties can be largely different from singly-connected ones such as quantum dots (QDs) or quantum disks. A comprehensive review on QRs and their features can be found in ref.^1^ (see also references therein for some other reviews on various aspects on the physics of QRs).

The practical realization of semiconductor QRs has been reported in a number of works. It is possible to find references on self-assembled InAs-GaAs or InAs-InP QR structures^2–10^ as well as on (mostly GaAs-based) droplet-epitaxy or molecular-beam-epitaxy ones^11–17^. Different image techniques used in those investigations reveal that in some cases the obtained doubly-connected complexes are actually not homogeneously shaped as circular nor do they have a constant rim height^15^. Instead, eccentrical geometries and single or multiple hill-like tops are noticed also including the formation of concentric double-ring (threefold-connected) configurations^16^. Oval-shape rings are shown in refs^5,8^ whilst asymmetrically lobed QRs appear in ref.^8^. Nonetheless, QRs with almost perfect circular or slightly oval shape were produced as well^4,7^.

From the theoretical point of view, the electronic states in QRs of arbitrary shape and non-uniform width were investigated in ref.^18^ with the inclusion of threading magnetic field effects. Studies on the Aharonov-Bohm effect, optical responses, spin-orbit interaction effects, influence of pressure and temperature, excitons, impurity states, and conduction band nonparabolicity in this kind of systems have been put forward^19–34^. Several other investigations dealing with deviations of the QR geometrical shape from perfect circular, homogeneous rim height or thickness have also been published^35–39^. In particular the dependency of electronic and optical properties on the shape eccentricity are discussed in refs^38,39^.

The influence of an externally applied intense laser field (ILF) on the electron states of quantum systems showing the discrete energy spectrum can be studied from the works by Gavrila^40–42^. In the particular situation of quantum semiconductor nanostructures, the intense laser field has a significant effect onto the electronic properties, thus affecting the optical transitions as well^43^. For instance, Cunha Lima *et al*. reported on the unexpected transition from single to double quantum well potential induced by ILF in a semiconductor quantum well^44^. More recently, the properties of the electron states under intense laser fields in circular and elliptical QRs^45^ and in double ring structures^46^ have been the subject of research.

As it is seen from cited literature, a lot of studies were devoted to different aspects of QRs. Nevertheless, the joint action of the ILF and strong magnetic field on eccentric QRs was not yet considered. Therefore, our work is focused on studying electron energy levels in an eccentric two-dimensional QR taking into account the effects of magnetic and intense nonresonant laser fields. After obtaining a set of analytical expressions for the ILF-related two-dimensional confining potential, the spectrum is obtained by solving the 2D effective mass partial differential equation with the use of the finite element method (FEM). A description of the theoretical environment is shown in section II. The section III is presented in two subsections; the first of them dedicated to show the external effects on electronic levels, and the second one to investigate the intensities of the intraband optical transitions with different polarizations of the incident laser fields. Finally, the section IV contains the conclusions.

## Theoretical Model

The model studied in this paper is that of an eccentric 2D GaAs-Al_0.3_Ga_0.7_As semiconductor quantum ring under the simultaneous actions of a non-resonant intense laser radiation and an externally applied magnetic field. The ring will be considered as a two-dimensional quantum structure of *D*_1_-symmetry class confined in the *x*–*y* plane. The effective mass and single particle approximations are used to solve the Schrödinger equation for the confined electron into the region of the heterostructure.

**Figure 1:**
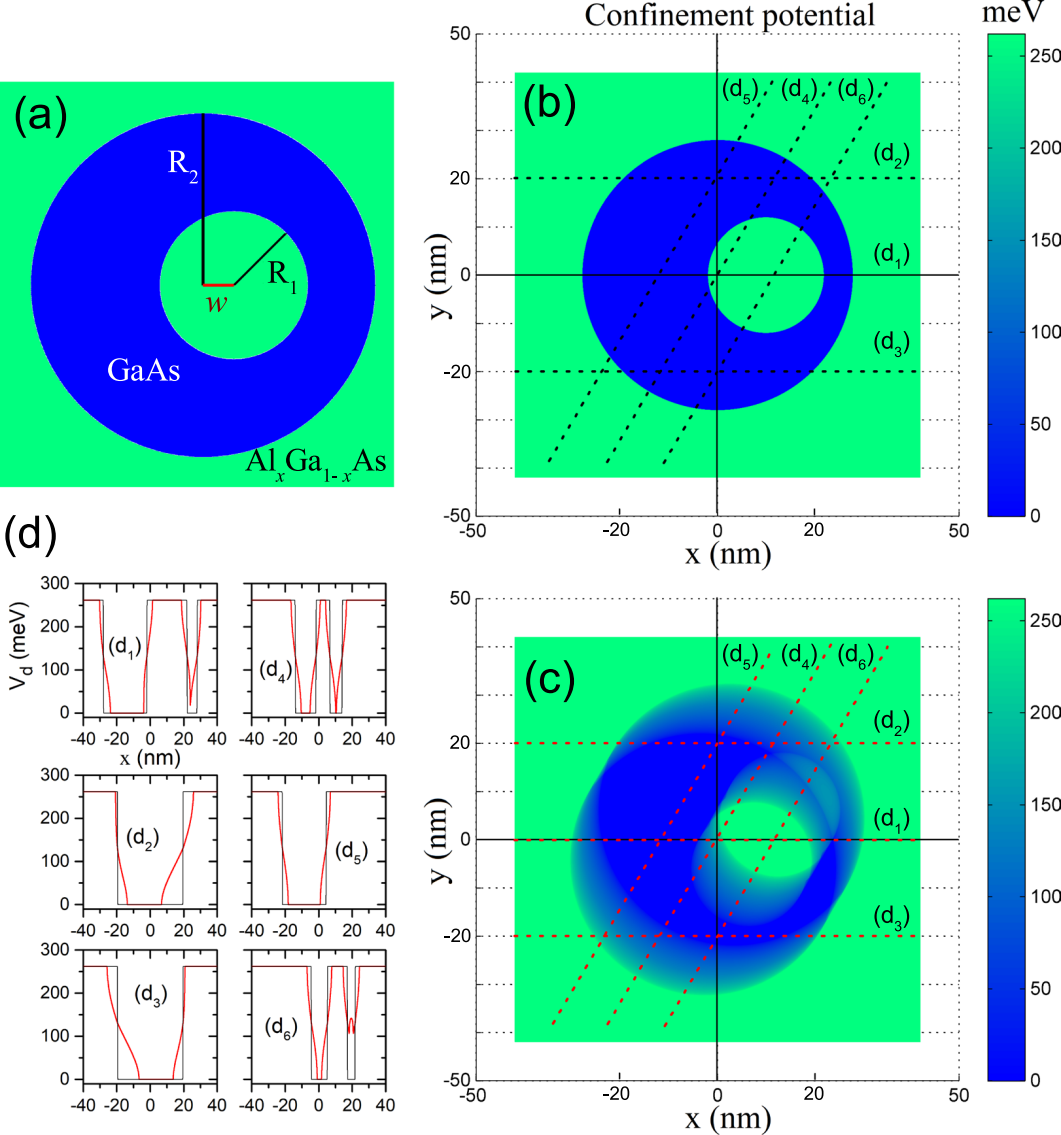
(**a**) A representation of the quantum ring geometry, (**b**) undressed confinement potential for *V*_0_ = 262 meV, *R*_1_ = 12 nm, *R*_2_ = 28 nm, and *w* = 10 nm, (**c**) laser-dressed confinement potential for *α*_0_ = 7 nm and *β* = *π*/3, and (**d**) 1D-functional dependence of the potential *V*_*d*_ along the special directions associated to the *d*_*n*_-lines indicated in (**b**,**c**) by black and red dashed lines, respectively.

The ring geometric parameters are the radii *R*_1_ and *R*_2_ of the circular inner and outer borders, respectively, and the *w*-eccentric displacement, defining the shift along the *x*-direction of the inner border (Fig. 1(a)). Between the two borders (inside the QR) the semiconductor material has a smaller band gap and, therefore, different electronic properties. Therefore we will further use the notations ${D}_{in}=\{(x,\,y)|{R}_{1}^{2}-{(x-w)}^{2}\le {y}^{2}\le {R}_{2}^{2}-{x}^{2}\}$ and ${D}_{out}=\{(x,\,y)|{R}_{1}^{2}-{(x-w)}^{2} > {y}^{2}\,{\rm{or}}\,{R}_{2}^{2}-{x}^{2} < {y}^{2}\}$ for the inside (blue area in Fig. 1(b)) and the outside (green area in Fig. 1(b)) plane domains of the QR, respectively. The non-resonant laser radiation is a linearly polarized monochromatic field that arrives perpendicularly to the ring-plane described by:

1 $${\overrightarrow{A}}_{l}(t)=(\hat{x}\,\cos \,\beta +\hat{y}\,\sin \,\beta )\,{A}_{0}\,\cos (\omega t),$$

where *A*_0_ and *ω* are the amplitude and frequency of the laser field’s vector potential ${\overrightarrow{A}}_{l}$, respectively, whereas *β* gives the orientation of the laser field polarization with respect to *x*-axis. Besides, $\hat{x}$ and $\hat{y}$ denote the in-plane unit vectors. The static external magnetic field will be considered perpendicular to the ring, i.e. $\overrightarrow{B}=B\hat{z}$, and we choose the symmetric gauge ${\overrightarrow{A}}_{m}=\frac{B}{2}(y\hat{x}-x\hat{y})$, with $\overrightarrow{B}=-\,\nabla \times {\overrightarrow{A}}_{m}$ ^47^. On the other hand, the QR electron confinement potential can be written as

2 $$V(x,\,y)=(\begin{array}{ll}\mathrm{0,} & {\rm{if}}\,(x,\,y)\in {D}_{in}\\ {V}_{0}, & {\rm{if}}\,(x,\,y)\in {D}_{out},\end{array}$$

where *V*_0_ is the conduction band discontinuity at the ring borders. Figure 1(b) presents the color gradient plot of this potential. The conduction band electron dynamics in the considered electromagnetic field is described by the two dimensional Schrödinger equation solution

3 $$\begin{array}{c}\{\hat{{\bf{p}}}+e[{{\bf{A}}}_{m}+{{\bf{A}}}_{l}(t)]\}\cdot (\frac{1}{2{m}^{\ast }(x,\,y)}\{\hat{{\bf{p}}}+e[{{\bf{A}}}_{m}+{{\bf{A}}}_{l}(t)]\})\psi (x,\,y,\,t)\\ \,\,\,\,+\,V(x,\,y)\,\psi (x,\,y,\,t)=-\,\frac{\hslash }{i}\frac{\partial }{\partial t}\,\psi (x,\,y,\,t),\end{array}$$

where m^*^ is the electron effective mass:

4 $${m}^{\ast }(x,\,y)=(\begin{array}{cc}{m}_{in}^{\ast }, & {\rm{if}}\,(x,\,y)\in {D}_{in}\\ {m}_{out}^{\ast }, & {\rm{if}}\,(x,\,y)\in {D}_{out},\end{array}$$

where $\hat{{\bf{p}}}=-\,i\hslash \nabla$ is the in-plane momentum operator, and *e* is the elementary charge. By using the Kramers-Henneberger unitary transformation^40–42,48^, Eq. (3) can be written as a time-independent equation:

5 $$\nabla \cdot [-\,\frac{{\hslash }^{2}}{2{m}^{\ast }(x,\,y)}\nabla \varphi (x,\,y)]-\,i\frac{eB\hslash }{2{m}^{\ast }(x,\,y)}(y\hat{x}-x\hat{y})\cdot \nabla \varphi (x,\,y)+\,[{U}_{d}(x,\,y)+{V}_{d}(x,\,y)]\varphi (x,\,y)=E\varphi (x,\,y\mathrm{).}$$

here, *U*_*d*_ is the laser-dressed diamagnetic term of the potential

6 $${U}_{d}(x,\,y)=\frac{{e}^{2}{B}^{2}}{8{m}^{\ast }}({x}^{2}+{y}^{2}+\frac{{\alpha }_{0}^{2}(x,\,y)}{2}),$$

with

7 $${\alpha }_{0}(x,\,y)=(\begin{array}{cc}{\alpha }_{0}, & {\rm{if}}\,(x,\,y)\in {D}_{in}\\ {\alpha }_{0}\frac{{m}_{in}^{\ast }}{{m}_{out}^{\ast }}, & {\rm{if}}\,(x,\,y)\in {D}_{out},\end{array}$$

and ${\alpha }_{0}=\frac{e{A}_{0}}{{m}_{in}^{\ast }\omega }\sim \frac{\sqrt{{I}_{0}}}{{\omega }^{2}}$ -with *I*_0_ representing the laser field intensity- is the laser parameter inside the ring^49^. The function *V*_*d*_(*x*, *y*) is the laser-dressed confinement potential^40–42^ which in this particular case can be analytically expressed by solving the integral ${V}_{d}=\frac{\omega }{2\pi }{\int }_{0}^{\frac{2\pi }{\omega }}\,V(X(t),\,Y(t))dt$ (the same integral form also lets us to deduce *U*_*d*_ in Eq. (6)), where *X*(*t*) = *x* + *α*_0_ cos *β* cos(*ωt*) and *Y*(*t*) = *y* + *α*_0_ sin*β* cos(*ωt*). By naming $\tilde{x}(x,\,y)=x\,\cos (\beta )+y\,\sin (\beta )$ and $\tilde{y}(x,\,y)=y\,\cos (\beta )-x\,\sin (\beta )$, one may obtain:

8 $${V}_{d}(x,y)={V}_{0}\,[1+{\bar{\sigma }}_{1}(\tilde{x}(x,y)-wcos(\beta ),\tilde{y}(x,y))+{\bar{\sigma }}_{2}(\tilde{x}(x,y),\tilde{y}(x,y))],$$

where ${\bar{\sigma }}_{i}(x,y)=({\sigma }_{i}(x,y)+{\sigma }_{i}(-x,y\mathrm{))/2}$ and we make use of the following notations:

9 $${\sigma }_{i}(x,\,y)=\frac{\mathrm{2(}-\,{\mathrm{1)}}^{i}}{\pi }\theta ({\alpha }_{0}(x,\,y)+x-{a}_{i}(\tilde{y}))Re\{\arccos \,(\frac{{a}_{i}(\tilde{y})-x}{{\alpha }_{0}(x,y)})\}$$

and

10 $${a}_{1}(\tilde{y})=Re\{\sqrt{{R}_{1}^{2}-{(\tilde{y}+w\sin (\beta ))}^{2}}\};\,{a}_{2}(\tilde{y})=Re\{\sqrt{{R}_{2}^{2}-{\tilde{y}}^{2}}\}\mathrm{.}$$

For practical purposes, *α*_0_(*x*, *y*) of Eq. (7) can be expressed by means of Heaviside step functions:

11 $${\alpha }_{0}(x,\,y)=({\alpha }_{0}\frac{{m}_{in}^{\ast }}{{m}_{out}^{\ast }}-{\alpha }_{0})[\theta (\sqrt{{x}^{2}+{y}^{2}}-{R}_{2})+1-\theta (\sqrt{{(x-w)}^{2}+{y}^{2}}-{R}_{1})]+{\alpha }_{0}.$$

It should be noted that the validity of the lowest order approximation (the zeroth order in 1/*ω*) is defined as $\hslash \,\omega \gg E({\alpha }_{0})$, which is called the high frequency condition, where *E*(*α*_0_) is the average excitation energy. Furthermore, there is no limitation on *α*_0_ apart from the high frequency condition. The details for dressed potential in Eq. (8) and the nonperturbative approach based on the Kramers-Henneberger translation transformation developed to describe the atomic behavior in intense high-frequency ILF can be found in refs^40–42,50–52^. Technically speaking, experimentally the ILF-effect can be implemented with an Argon laser, commercially available, which satisfies the condition that its emission energy is much higher than the energy of the confined states reported in this study.

Solution of the Eq. (5) must be carried out numerically. However, in the limiting case corresponding to a quantum disk (*R*_1_ = 0), it has an analytical form and the transcendental equation with Ben-Daniel Duke boundary conditions to calculate the energy eigenvalues with finite confinement potential and radius *R*_2_ is given by^53^:

12 $$\frac{1}{{m}_{in}^{\ast }}\,\alpha [{J}_{m-1}(\alpha {R}_{2})-{J}_{m+1}(\alpha {R}_{2})]\,{K}_{m}(\beta {R}_{2})=-\,\frac{1}{{m}_{out}^{\ast }}\beta [{K}_{m-1}(\beta {R}_{2})+{K}_{m+1}(\beta {R}_{2})],$$

where $\alpha =\sqrt{\frac{2{m}_{in}^{\ast }}{{\hslash }^{2}}\,{E}_{mn}}$, $\beta =\sqrt{\frac{2{m}_{out}^{\ast }}{{\hslash }^{2}}\,({V}_{0}-{E}_{mn})}$, *J*_*m*_(*x*) is the Bessel function of the first kind, *K*_*m*_(*x*) is the Bessel function of second kind, *m* = 0, ±1, ±2, ±3, ..., and *n* = 1, 2, 3, .... Such equation will be used to test our results in the limiting case of an isolated quantum disk.

The color gradient plot of Eq. (8) is presented in Fig. 1(c). One may notice that the *D*_1_-type symmetry of the confinement potential is destroyed by laser dressing excepting the cases *β* = *nπ*/2, where *n* is an integer. We note that the applied magnetic field is not going to alter the symmetry of the system. In Fig. 1(d) we represent the 2D-images containing the shape of the confinement potential along the special directions depicted in panels 1(b) and 1(c). The black lines correspond to the potential in panel 1(b), without ILF-effects, while the red lines correspond to the potential in panel 1(c) where the ILF-effects have been included with *α*_0_ = 7 nm and *β* = *π*/3. From the figures portraying the distinct *d*_*n*_ (*n* = 1, ..., 6) projections some specific comments are worthy: (*i*) for energies below the half-height of the potential barrier (*V*_0_/2) there is an effective decrease of the quantum well widths, while for energies greater than the half-height of the potential barrier the effective sizes of the quantum wells actually increase, (*ii*) in those special directions where a double well structure appears (*d*_1_, *d*_4_, and *d*_6_), the presence of ILF induces a coupling between the wells–this effect is associated both to the drop of the barrier height and the decreasing of the effective width of the central barrier, (*iii*) note the appearance of a double quantum well structure located on the right side of panel *d*_6_, which corresponds to the phenomenon previously explained in the above mentioned work by Cunha Lima *et al*.^44^ and finally (*iv*) the Fig. *d*_3_ is obtained from Fig. *d*_2_ by making a reflection with respect to *x* = 0.

**Figure 2:**
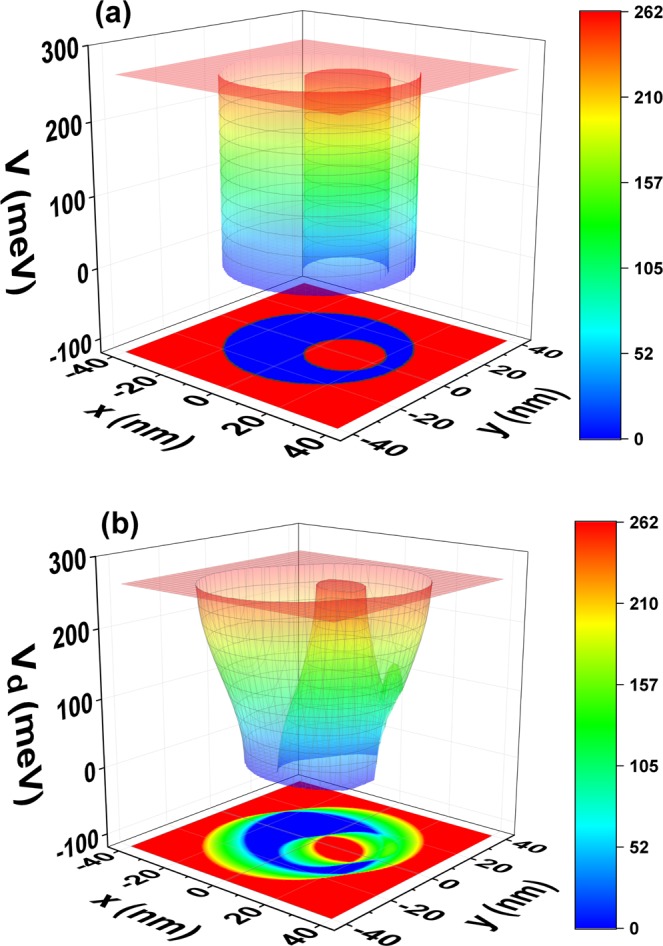
(**a**) The undressed confinement potential with *xy*-plane projection for a GaAs-Al_0.3_Ga_0.7_As eccentric QR; and (**b**) the laser-dressed potential confinement with *xy*-plane projection.

For clarity purposes, a 3D-color gradient for the undressed and dressed-laser potential is presented in Fig. 2. The panels 2(a) and 2(b) are 3D-views of the panels 1(b) and 1(c), respectively. The color scales vary from the zero value in the potential, corresponding to the blue color, to its maximum value of 262 meV, represented by the red color. The transparent design lets us to observe the complex shape of potential when the laser is turned on in Fig. 2(b).

By solving Eq. (5) one may obtain the eigenvalues and eigenfunctions of the confined electron into the region of the eccentric quantum ring under the perpendicular applied magnetic field and ILF-effects. We denote by *ϕ*_*j*_ and *E*_*j*_ the normalized eigenfunction and eigenvalue corresponding to *j*^th^ electronic level in the conduction band. With the information for the spectrum, we shall further consider the intraband absorption transitions that may occur from the ground state to some of the electronic excited levels. The transition energies are labeled as Δ*E*_*j*1_ = *E*_*j*_ − *E*_1_ and the related intensities of the optical response will be calculated for *ξ*-polarization of the incident resonant light from a probe laser (where the options *ξ* = (*x*, 0) and *ξ* = (0, *y*) are considered in the present work) via the expression

13 $$I \sim {|\iint {\varphi }_{j}^{\ast }(x,y)\xi {\varphi }_{1}(x,y)dxdy|}^{2}.$$

## Results and Discussion

### External fields effects on the electron energy levels

To perform out calculations we have considered a common pair of semiconductor materials, GaAs/Al_0.3_Ga_0.7_As. We used the following numerical values^11^: *V*_0_ = 0.262 eV, ${m}_{in}^{\ast }=0.067\,{m}_{0}$, and ${m}_{out}^{\ast }=0.093\,{m}_{0}$, where *m*_0_ is the free electron mass. Eq. (5) is numerically solved by the FEM using a 2D model in COMSOL Multiphysics software^54^. The Schrödinger equation has been put in the general partial differential equation (PDE) form. The eigenvalue solver was used with a zero-flux boundary condition. The boundary was taken to be a circle with radius equals to 2*R*_2_. In our calculations we used an extra refined mesh with a number of degrees of freedom $\sim 50000$. By adapting parameters numerical values to those used in ref.^45^ we were able to reproduce the results reported by Chakraborty *et al*. for the circular ring case. The observed differences between our solutions and those in ref.^45^ are not larger than 1 meV, for all values of the ILF-parameter and the magnetic field, which in ref.^45^ was maximum 20 T. However, in the present study the magnetic field strength will be extended up to 30 T, in consonance with experimental values available in the literature^55^. The outer border of the QR was fixed at *R*_2_ = 28 nm. Then, the number of electron energy levels in the conduction band is further determined by *R*_1_.

**Figure 3:**
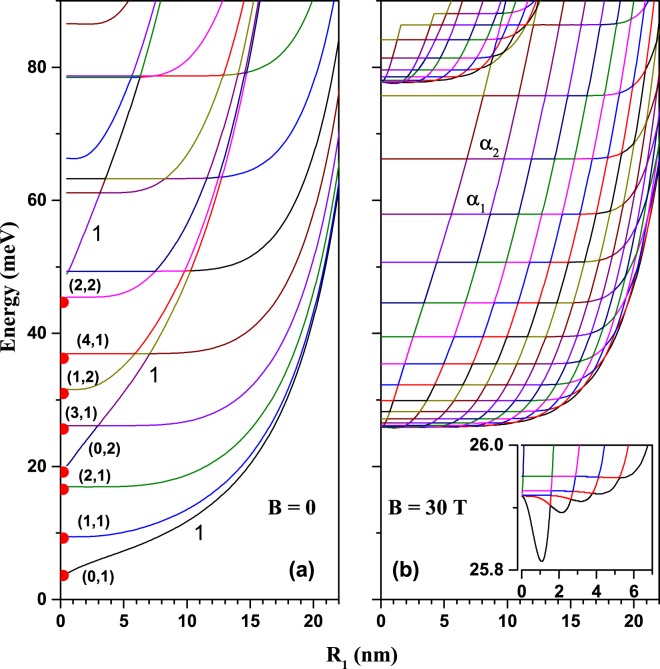
(**a**) The lowest thirty allowed energy levels of a GaAs-Al_0.3_Ga_0.7_As concentric QR (*w* = 0)in the absence of external fields, as functions of the inner border radius (several eigenstates are twofold degenerated -see discussion in the text). The curves are labeled by the pair (*m*, *n*) (see also the text); (**b**) the results are the same as in (**a**) but for an applied magnetic field (all degeneracies are lifted) and *α*_0_ = 0. The inset in (**b**) represents the lowest levels energies for small *R*_1_. The results are for fixed *R*_2_ = 28 nm. Solid symbols near to the vertical axis in Fig. 3(a) are the eigenvalues for a single quantum disk with *R*_2_ = 28 nm and *R*_1_ = 0 obtained by using Eq. (12) .

Figure 3(a) shows the first thirty low-lying levels as functions of the inner border radius *R*_1_. Calculation was done for the concentric GaAs-Al_0.3_Ga_0.7_As QR in the absence of the ILF-effects (*α*_0_ = 0). The ground state is non-degenerated, corresponding to a *s*-like orbital. The second non-degerate state corresponds to a *s*-like one, related to another energy sub-spectrum with a different value of the *m*-quantum number (see Eq. (12)). It can be seen as the first high-slope line that crosses several excited levels. We use label “1” to point at the positions of *s*-like states. So,a third level of that kind appears as it is indicated. Our results follow the same tendency as those reported in ref.^27^. All other levels in Fig. 3(a) are doubly-degenerated. At the left extreme of Fig. 3(a), for small values of *R*_1_, the spectrum will correspond to that of a quantum disk. The full symbols close to the vertical axis of the figure correspond to the analytical calculation obtained by means of the transcendental Eq. (12)^53^. Such equation is appropriate to obtain the limiting case when *R*_1_ = 0. It is observed that our numerical results are in excellent correspondence with those analytical solutions. The notation in parenthesis shown for the lowest eight states, represents the pair of quantum numbers (*m*, *n*), in relation with the discrete spectrum of a quantum disk. There, *n* corresponds to *n*^*th*^ zero of the transcendental equation for a fixed value of *m*. The observation of several sub-spectra related to the *n* number is in agreement with the calculations made by LLorens *et al*.^56^ for this kind of systems. The increasing character of the energies as *R*_1_ goes from zero to 22 nm is due to the magnification of the confinement due to the reduction of the ring’s area, $\pi \,({R}_{2}^{2}-{R}_{1}^{2})$. Notice that the excited energy levels are almost constant up to specific value of ${R}_{1}^{L}$, which increases as the energy levels are higher. In the range $0 < {R}_{1} < {R}_{1}^{L}$ the results correspond to typical QRs energy spectra. At the right extreme of the Fig. 3(a) with *R*_1_ → *R*_2_ the spectrum resembles a quasi-one dimensional quantum wire system.

The Fig. 3(b) presents the results obtained under the same conditions for the structural parameters as in Fig. 3(a), but in the presence of an applied magnetic field (*B* = 30 T). It is observed that the number of the discrete states per unit energy increases due to the parabolic-type magnetic potential in Eq. (6). In the same way as it was described in Fig. 3(a), in this case it is observed that the energy levels have a range of *R*_1_ ($0 < {R}_{1} < {R}_{1}^{L}$) where they behave as constants. Then, from ${R}_{1} > {R}_{1}^{L}$, each state presents a high rate of growth in energy. Besides, ${R}_{1}^{L}$ increases as the order of the state under consideration grows. For example, for the state labeled with *α*_1_, ${R}_{1}^{L}=15.7$ nm and for the state labeled with *α*_2_, ${R}_{1}^{L}=16.7$ nm. This behavior leads to a very characteristic multiple-crossing aspect of the conduction band. It is also visible that for relatively small values of *R*_1_ the low-lying energy levels are strongly increased by the magnetic field in comparison with Fig. 3(a), although for large *R*_1_ values the increase is less obvious. A second sub-spectrum appears close to 75 meV and it is a consequence of the axial symmetry of the QR. The inset in Fig. 3(b) shows the first five energy levels for small *R*_1_. The oscillations observed for the ground state are of Aharonov-Bohm type, due to the magnetic field effect. This means that, for example, the ground state changes its symmetry from *s*-type to *p* (or *d*)-type state as a function of *R*_1_. At this point, it is worth to highlight that, for further calculations in this paper, we choose to fix the value of the inner radius at *R*_1_ = 12 nm.

**Figure 4:**
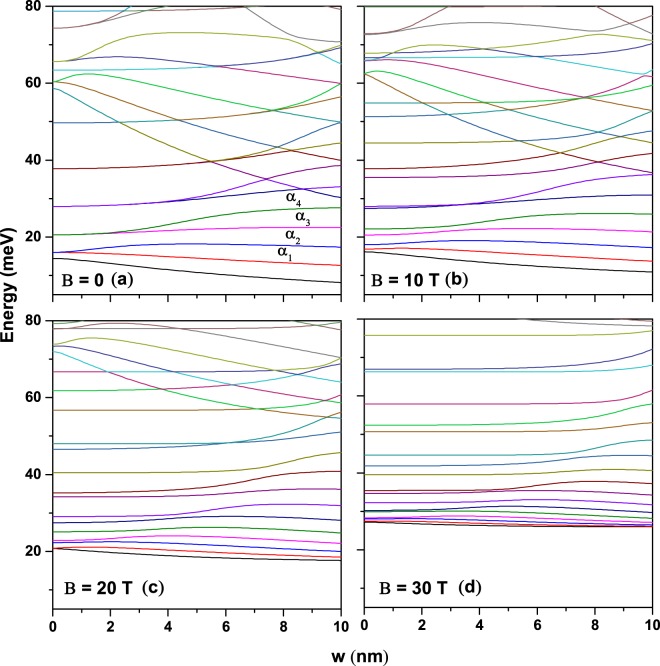
Eccentricity effect on the low-lying energy levels in the absence of laser field for several values of the applied magnetic field. Results are for a GaAs-Al0.3Ga_0.7_ As eccentric QR with *R*_1_ = 12 nm and *R*_2_ = 28 nm.

For fixed *R*_1_ and *R*_2_ values, we investigate the effect of the eccentric *w*-displacement on the conduction band structure. Figure 4(a) presents the energy levels as functions of *w* in the absence of external fields. One may observe that the eccentricity splits all degenerated levels into two ones, since the circular symmetry of the ring is altered. Such a degeneracy breaking takes place at larger values of the *w*-parameter as the order of the excited levels increases. The ground state energy is a decreasing function of the *w*-parameter due to the increasing of the effective area of the region where the electron is confined. Note that the lowest two non-degenerated levels, produced by the symmetry breaking, are generally decreasing functions of *w*, whereas the higher excited levels are generally increased by the growth of the asymmetry. The *α*_1_ state has a *p*_*x*_-symmetry when *w* = 0 and behaves in a similar way to *s*-type states when the symmetry is broken; i.e., it decreases when *w* augments. In the case of the *α*_2_-state, for zero and finite values of the *w*-parameter, it has a *p*_*y*_-symmetry and its energy increases due to the decreasing of the spatial extension of the region over which the state extends (for *w* finite values, the *α*_2_-state is pushed to the left where the *y*-extension of the structure decreases due to the circular shape of the outer radius of the ring). The increasing character in the energy of the *α*_3_ and *α*_4_ states, which have *d*-symmetry, is also explained by the breaking of the axial symmetry of the QR and by the decrease in the vertical distance between its lobes as they are pushed towards the left-hand part of the ring. Note the presence of the *n* = 2 sub-spectrum with energies larger than 55 meV at *w* = 0 for which the energy levels physics is the same as for *n* = 1. Figure 4(b–d) show the same kind of results without ILF-effects but for different values of the externally applied magnetic field. It is observed that the magnetic field produces an overall rising of the allowed energy values as well as a decrease of the interlevel energy difference, and also prevents the crossings of the low-lying levels, this effect is due to the extra magnetic field confinement. From Fig. 4(d) one may conclude that in the strong magnetic field regime the conduction band structure is less sensitive to the asymmetry induced by the eccentric *w*-parameter; i.e., in this case the breaking of the symmetry can be treated as a perturbation on the system, with the magnetic field being the dominant factor.

**Figure 5:**
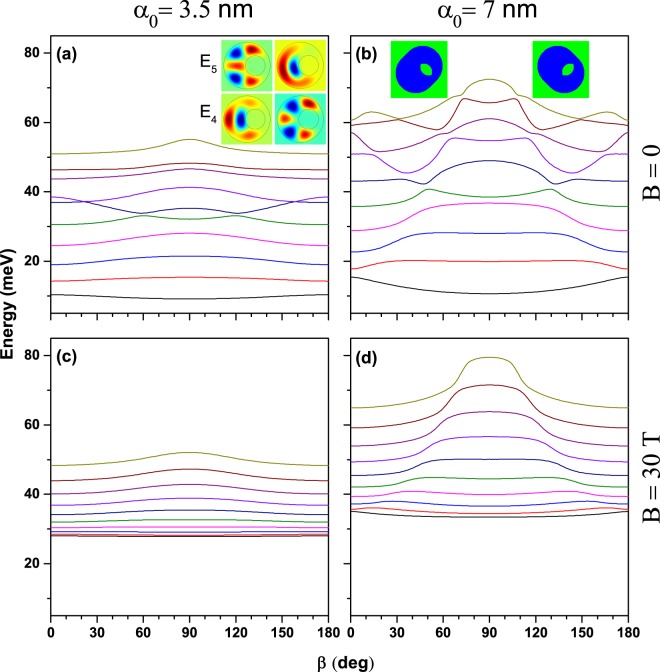
Effect of intense laser field incident direction (*β*) on the low-lying energy levels for different values of the external magnetic field. The results correspond to a highly eccentric QR with *w* = 10 nm in a GaAs-Al_0.3_ Ga_0.7_ As structure with *R*_1_ = 12 nm and *R*_2_ = 28 nm. See text for the description of inset contents.

We have also investigated the influence of the ILF polarization direction on the energy spectrum of the eccentric QR. In particular, our calculation has focused on the first ten energy levels in a highly eccentric system, for different values of the applied magnetic field and ILF-parameter. It is clear but not obvious that all energy levels are even functions of the *β*-angle with respect to *β* = 90° (see inset in Fig. 5(b), which corresponds to a dressed potential *V*_*d*_ for values *β* = 45°, 135°, with *α*_0_ = 7 nm). Figure 5(a,b) show the results obtained for *B* = 0. As it was observed in Fig. 4(a), with *w* = 10 nm, the energies of the ten lowest states are smaller than *V*_0_/2 = 131 meV. In consonance with Fig. 2(b), for finite values of the *α*_0_-parameter -which induces a narrowing of the potential profile-, these ten states should exhibit a blue shift, as it is actually depicted in Fig. 5(a,b). At the ILF-parameter *α*_0_ = 3.5 nm Fig. 5(a), the effect of the laser polarization rotation is quite interesting, producing a slow increase of the energies (except for the those of *s*-like states). For the larger value of the ILF-parameter, the further asymmetry introduced by laser-dressing becomes more obvious when increasing the value of *β*. Figure 5(b) also illustrates the complex behavior of the conduction band with the rotation of laser polarization. Several anti-crossings between higher excited levels can be observed. They are also present in Fig. 5(a), but become less evident and locate closer to the *β* = 90° symmetry point of the curves. The inset in Fig. 5(a) shows the exchange of symmetry between the fourth and fifth excited states at the anti-crossing point occurring for *β* = 120°.

Figure 5(c,d) present the ILF rotation effect in the strong magnetic field regime. For the smaller value of *α*_0_, the values of the lowest energy levels are almost unaffected showing that the magnetic field has the dominant effect. The interlevel energies are very slowly increasing with the order of the energy levels (note in 5(c) for instance that *E*_9_ − *E*_8_ > ... > *E*_2_ − *E*_1_ > *E*_1_ − *E*_0_, where *E*_0_ is the ground state). We may conclude that for relatively weak ILF-parameter, in the strong magnetic field regime the eccentric ring energy levels are practically invariant under the laser polarization rotation. However, for larger values of *α*_0_ (Fig. 5(d)) the highly excited energy levels are more sensitive to the laser polarization rotation. Multiple anti-crossings occur in the strong magnetic field case. Comparing Fig. 5(a,c) we note that in the presence of the applied magnetic field all the states are concentrated in a range of 20 meV with gravity centre at 40 meV (the lower/higher states increase/decrease their energies); whereas for the configurations corresponding to Fig. 5(b,d), the magnetic field acts by inducing a blue-shift for all the states.

**Figure 6:**
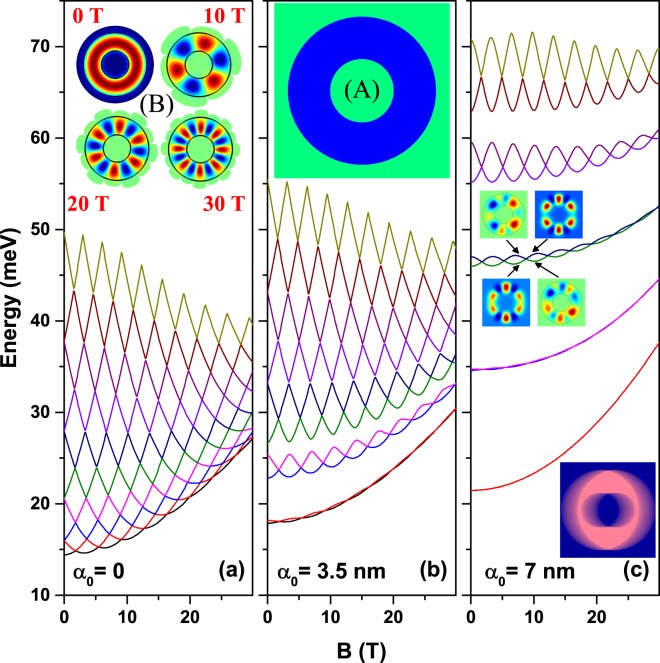
The first ten electron energy levels in a GaAs-Al_0.3_Ga_0.7_As two-dimensional quantum ring as functions of the perpendicularly applied magnetic field for three values of the intense laser field parameter with *x*-polarization (*β* = 0). The results are for a non-eccentric circular quantum ring (see inset (A) in Fig. (**b**)). Inset (B) in Fig. (**a**) shows the wave functions for the ground state for *α*_0_ = 0 and *B* = 0,10,20,30 T. The geometric setup of the structure is *R*_1_ = 12 nm, *R*_2_ = 28 nm, and *w* = 0. The inset in (**c**) shows the laser-dressed confinement potential in color gradient plot.

We are particularly interested in observing the changes of the confined conduction states induced by the continuous variation of the magnetic field and laser parameter. So, we start by presenting the concentric (non-eccentric) QR case where some of the energy levels are degenerated in the absence of external fields (*B* = 0 and *α*_0_ = 0), on the vertical axis, and then connect the externally applied fields. Consequently, Fig. 6 shows the first ten energy levels for an electron confined in a GaAs-Al_0.3_Ga_0.7_As QR as function of the perpendicular applied magnetic field for three values of the laser parameter. For *α*_0_ = 0 there is no in-plane asymmetry and the degenerated levels are split by a Zeeman-like effect (Fig. 6(a)). The higher the degenerate energy, the stronger the split will be, as a direct consequence of higher values of the *m*-quantum number (see discussion of Fig. 3). This behavior creates the multiple crossing pattern observed in this figure; the crossings can be seen as accidentally degeneracy between energy levels. As a consequence of the crossings the energy levels are oscillating with *B*. One may observe that some particular energy level oscillates and also changes the symmetry of its wave function. For example, the ground energy level, which exhibits a clear oscillation pattern, is globally increasing with *B* in a parabolic manner with oscillations passing from *s*-like symmetry of the wave function for *B* = 0 to many-maxima aspect for higher values of the magnetic field (see inset labelled as (B) in Fig. 6(a)). The envelope quadratic function which describes the energy of the ground state, in meV, is given by the fit *E*_0_ = 14.39 + 0.06*B* + 0.0124*B*^2^. Using the symmetry of the quantum disk, in Fig. 6(a) at *B* = 0, 10, 20, *and* 30 T the pairs of quantum numbers (*m*,*n*) for the ground state are (0, 1), (3, 1), (6, 1), and (8, 1), respectively. This is the reason for the number of antinodes in the inset (B) is 0, 6, 12, and 16 as the magnetic field increases.

Figure 6(b,c) show how the above described behavior changes when the laser field is added to the model. The entire band structure is expanded along the energy axis and degeneracy is broken even for *B* = 0. Anti-crossings occur, beginning at lower energy levels for relative small laser parameter (Fig. 6(b)) and continuing with higher energy levels as *α*_0_ increases (Fig. 6(c)). Energy levels oscillate in pairs with reduced amplitude for low-lying levels and increased amplitude for highly excited levels. In the strong laser field studied, energy levels are generally blue-shifted by the increase of the magnetic field, this effect being more obvious for lowest levels. In Fig. 6(b) the quasi-degenerated ground state can be associated to a situation with two interacting quantum dots, whereas in Fig. 6(c), for very large value of the ILF-parameter, it turns out as a two isolated quantum dot configuration. Then, the ILF-effect lies in the division of the space into two well defined quantum dot regions, as shown in the inset in Fig. 6(c). We have to stress that in the Fig. 6(b,c) there appears the degeneracy between states that conform a pair (1–2, 3–4, 5–6, …, etc). Additionally, and due to the broken symmetry at *B* = 0, which is associated with the ILF-parameter different of zero, the mixing between states with different values of the *m*-quantum number, we can observe the anti-crossing behavior between states which belongs to different pairs (2–3, 4–5, 6–7, …, etc). This behavior is more evident as the asymmetry of the ring increases, which can be seen by comparing Fig. 6(b,c).

**Figure 7:**
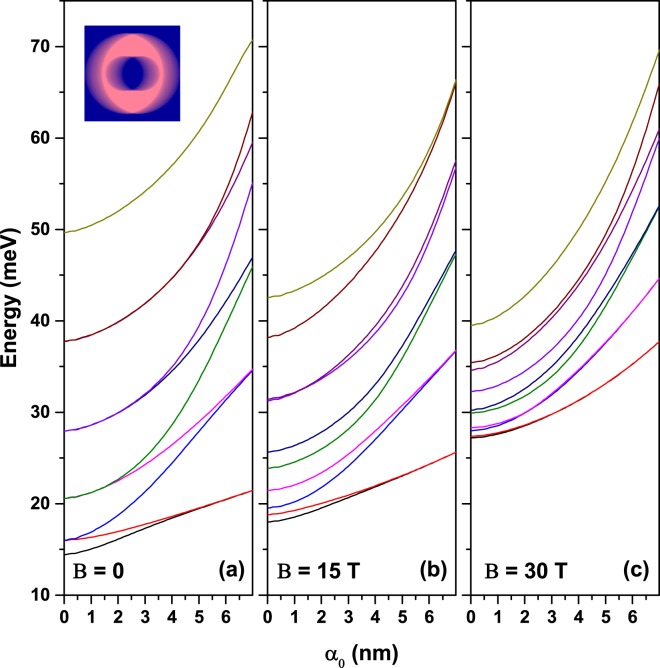
The first ten energy levels in a GaAs-Al_0.3_Ga_0.7_As QR as functions of the ILF-parameter for three values of the perpendicularly applied magnetic field. Results are for concentric (non-eccentric) QR and *x*–polarization of the laser field (*β* = 0). The structure geometric parameters are *R*_1_ = 12 nm, *R*_2_ = 28 nm, and *w* = 0. The inset shows the laser-dressed confinement potential in color gradient plot.

It may be also instructive to consider the behavior of the energy levels in a concentric QR with respect to the ILF-parameter for different applied magnetic field values. In the results presented in Fig. 7 *α*_0_ was continuously modified between zero and 7 nm. Figure 7(a) shows the ones obtained in the absence of magnetic field. The lowest calculated level is non-degenerate but all others are doubly degenerated for *α*_0_ = 0. This is due to the rotational symmetry of the QR. Increasing *α*_0_ leads to the splitting of degenerate levels as a consequence of the laser-induced symmetry breaking. All energies augment with *α*_0_, the increment being larger in absolute value for highly-excited levels. The increasing behavior of the energy levels with respect to the variation of the *α*_0_-parameter is due to the narrowing of the potential profile for energies below *V*_0_/2. Note that the maximum energy of all depicted states in Fig. 7(a) is 70 *meV* < *V*_0_/2. The splitting of degenerate levels is stronger for lower-lying levels and occurs only for relatively high values of the laser parameter for highly excited levels. The inset in Fig. 7(a) shows the color gradient plot of the confinement potential at *α*_0_ = 7 nm and reveals that the QR circular symmetry was transformed by laser-dressing into a configuration of two QD-like structures. This behavior explains why, for large *α*_0_ values, the energy levels redistribute pairwise: 1–2, 3–4, 5–6, etc., and become either symmetric or antisymmetric states. Figure 7(b,c) present the same kind of results but for medium and high magnetic field values. The degeneracy is broken even for *α*_0_ = 0 due to Zeeman-like effect. The entire group of energy levels is compressed on vertical axis and the laser induced absolute shift in energy is augmented by the presence of the magnetic field.

**Figure 8:**
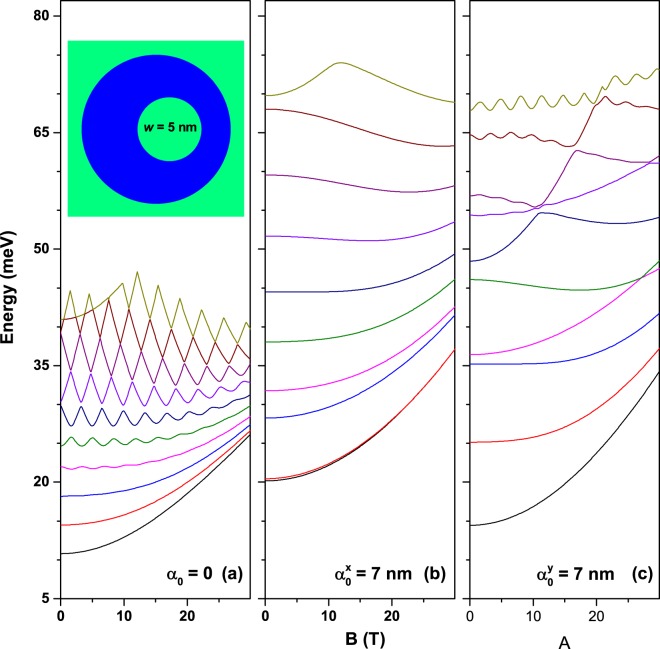
The first ten energy levels in a GaAs-Al_0.3_Ga_0.7_As QR as a function of the applied magnetic field for small eccentric parameter: (**a**) without laser field; (**b**) with laser field polarized in *x*–direction (*β* = 0); (**c**) with laser field polarized in *y*–direction (*β* = *π*/2). The structure geometric parameters are *R*_1_ = 12 nm, *R*_2_ = 28 nm, and *w* = 5 nm.

We further investigated the effect of the magnetic field on the energy levels in eccentric QRs with and without laser field. This case exhibits a more complex behavior since three different types of asymmetry occur: magnetic-field-induced, laser-induced, and geometrical. Results presented in Fig. 8 are for medium eccentric parameter (*w* = 5 nm). Figure 8(a) shows a very interesting change of the oscillating behavior of levels by passing from ground state to highly excited states in the absence of the laser field. The first several low-lying levels are oscillating with the magnetic field at small amplitude values. Then, by going to higher energy levels one may observe that oscillations increase in amplitude and finally they lead to the kind of crossing pattern discussed above for Fig. 6. This transition is entirely due to the geometric asymmetry of the confinement potential (see inset). In fact, eccentricity tends to quench the Ahoronov-Bohm oscillations, resembling the effect of the application of a constant electric field to the system. The low-lying levels are more affected by the broken symmetry and under the magnetic field they behave as typical anti-crossing states. The high excited levels are less affected by the geometric asymmetry and therefore they still exhibit the characteristics of degenerate levels. At zero magnetic field they couple in pairs with almost the same energy and Zeeman-like splitting for increasing magnetic field. This explains the slow but obvious transition from anti-crossing behavior at small energies to crossing behavior for higher energies. Figure 8(b,c) present the same calculation for *x*-polarized and *y*-polarized laser-dressing, respectively. From Fig. 8(b) it is possible to observe that oscillating behavior completely disappears because the laser field enhances the asymmetry produced by eccentric confinement. As a result all degeneracies are broken including those of highly excited levels. Figure 8(c) shows a somewhat more complicated effect of the magnetic field on the energy levels. Some oscillations are visible for highly excited states which indicates that laser-dressing in *y*-direction partially restore the symmetry broken by eccentric geometry.

**Figure 9:**
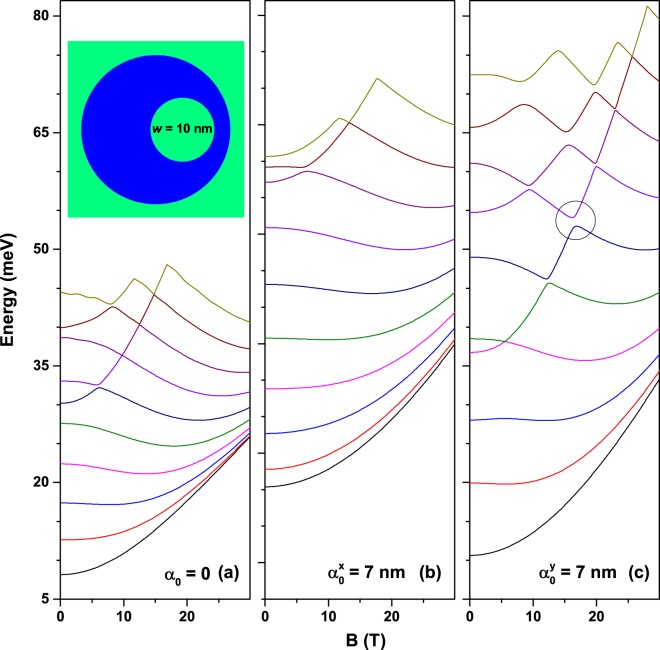
The same as Fig. 8 for large eccentric displacement.

Figure 9 contains the results for a highly eccentric QR with *w* = 10 nm; organized similarly to those in Fig. 8. One may observe from Fig. 9(a) that oscillations are not present as in Fig. 8(a), the obvious reason being the complete lifting of degeneracy induced by a highly asymmetric geometry. However, some excited levels are still crossing each other. As compared with Figs 8(b) and 9(b) shows a more pronounced increase of the energy levels for augmenting magnetic field strength. However, the general coalescent aspect of the levels with the increase of *B* is preserved. In addition, Fig. 9(c) shows some anti-crossings between levels but contrary to Fig. 8(c) no clear oscillator behavior may be observed. This could be explained by the fact that geometric asymmetry is so important that laser field in this range is not able to restore a magnetic-like behavior.

### Intraband optical transitions under magnetic and laser fields

In this subsection we investigate the transition energies and intensities associated to intraband absorption of linearly polarized light. We only take into account transitions from the ground level to several excited levels. This approach is justified when only the first level is significantly populated which may be the low temperature case. Calculations were made for nine excited levels and transition energies are plot as functions of the magnetic field for different configurations of the laser field. Transition intensities are estimated in arbitrary units by using Eq. (13) with a natural logarithmic re-scaling:

14 $$I=\,\mathrm{ln}\,({|\iint {\varphi }_{j}^{\ast }\xi {\varphi }_{1}dxdy|}^{2}+1)\,.$$

The intensity as given by Eq. (14) will be double-coded on the plots: by linewidth and by color. Thin and red line will represent very weak, near zero absorption. Thick and blue line will represent very strong, near maximum absorption.

**Figure 10:**
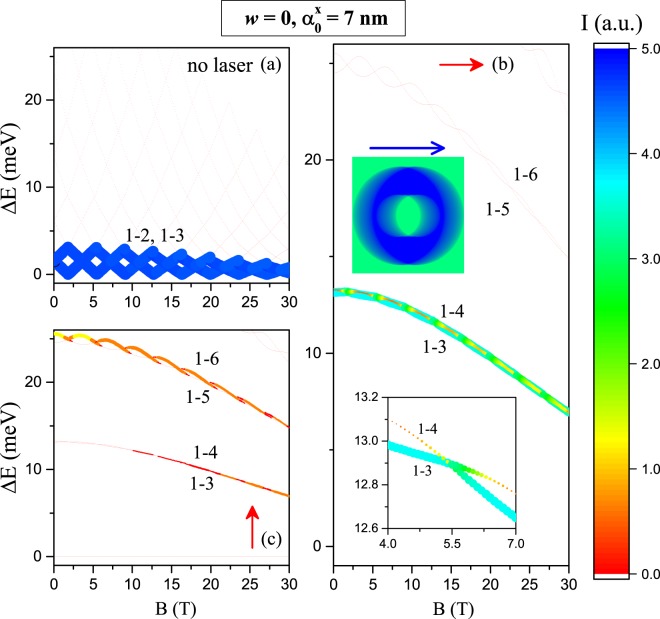
Magnetic field effect on the *ϕ*_1 _→ *ϕ*_*j*_ allowed intraband optical transitions in a GaAs-Al_0.3_Ga_0.7_As concentric QR (*w* = 0) (where *ϕ*_1_ and *ϕ*_*j*_ correspond to the ground and excite states wavefunctions, respectively, with *j* = 2, 3, 4, ...): (**a**) without effects of nonresonant intense laser field, for any polarization of the absorbed light from a probe laser; (**b**) with *x*-polarized nonresonant laser field dressing and *x*-polarized absorbed light from a probe laser; (**c**) with *x*-polarized nonresonant laser field dressing and *y*-polarized absorbed light from a probe laser. The vertical axis represents transition energy and the logarithmic intensity is simultaneously coded in the line width and color: 0–red is forbidden and corresponds to zero intensity of the optical transition; 5−blue is allowed and corresponds to maximum intensity of the optical transition (see the color bar). The inset represents the color gradient plot of the *x*-polarized (*β* = 0) laser-dressed confinement potential. The structure geometric parameters are *R*_1_ = 12 nm and *R*_2_ = 28 nm. The color bar is valid in all the figure.

Figure 10 presents the results for concentric QR with and without laser field dressing. One may observe that in the absence of the nonresonant laser field (Fig. 10(a)), the transitions 1–2 and 1–3 are very intense but with low transition energy (<3 meV). All other transitions are forbidden, being the QR practically transparent for optical radiation. The zero transition energies in the figure correspond to transitions between accidentally degenerated states in Fig. 6(a). Figure 10(b,c) present absorption in the presence of the laser dressing. In Fig. 10(b) the resonant incident radiation is polarized along the *x*-direction of the heterostructure -parallel polarization (see inset)- and in Fig. 10(c) it is polarized along the *y*-direction -perpendicular polarization. In the parallel polarization case (Fig. 10(b)), the transitions 1–3 and 1–4 occur with relatively high intensity and higher energy transitions 1–5 and 1–6 are almost forbidden. It may be observed that allowed transitions are red-shifted with the increase of magnetic field and some intensity oscillation occur, simultaneously with slow decrease of the absorption (see the inset in Fig. 10(b)). In the perpendicular polarization case (Fig. 10(c)) transitions 1–3 and 1–4 are almost forbidden and transitions 1–5 and 1–6 are allowed for relatively weak magnetic field and are significantly red-shifted and almost vanish for large values of *B*. Transitions 1–3, 4 are at the limit of the far infrared domain (~2 THz) and transitions 1–5,6 are middle infrared. The horizontal transitions at Δ*E* = 0 in Fig. 10(b,c) are due to the two fold degenerated ground state.

**Figure 11:**
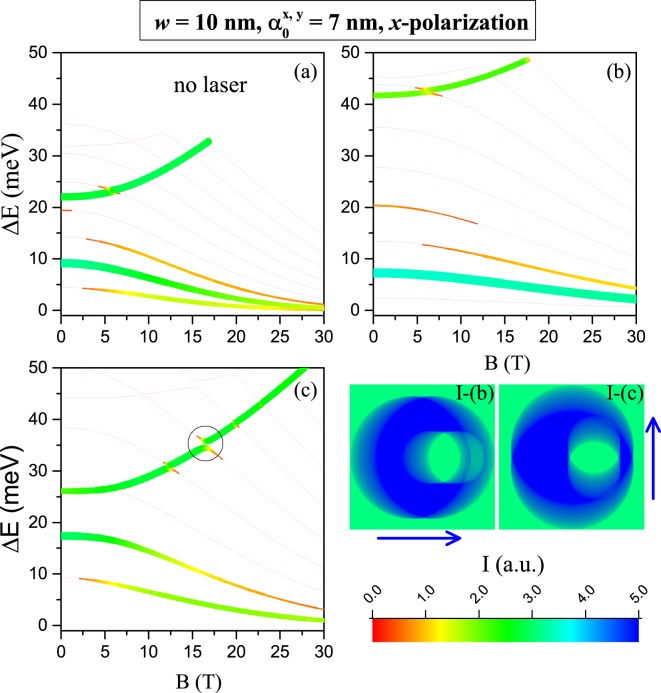
Magnetic field effect on the *ϕ*_1 _→ *ϕ*_*j*_ allowed intraband optical transitions in a GaAs-Al_0.3_Ga_0.7_As eccentric QR-*w* = 10 nm (where *ϕ*_1_ and *ϕ*_*j*_ correspond to the ground and excite states wavefunctions, respectively, with *j* = 2, 3, 4, ...). Results are for *x*-polarized incident resonant light from a probe laser: without nonresonant laser field (**a**); for *x*-polarized nonresonant laser field dressing (**b**); and for *y*-polarized nonresonant laser field dressing (**c**). The representation style (color code and widths of the lines) and insets are the same as in Fig. 10.

Figure 11 shows the intraband transition energies and intensities for an eccentric QR, where absorbed light is polarized in the eccentricity direction (*x*). Two cases are considered: laser dressing on *x* and *y*-axis. It may be observed that only a few transitions are allowed (keep in mind that red lines are for *I* → 0 whereas the blue ones are for the maximum calculated dipole moment values). Generally the high energy transitions are blue-shifted by the increase of magnetic field and keep their intensities almost constant. Near to *B* = 16.5 T and Δ*E* = 35.82 meV appears the anti-crossing highlighted by the circle in the Fig. 11(c). Such anti-crossing shows up as a symmetry interchange between the wave functions and consequently the growing energy transition associated to the green line in Fig. 11(c) -with almost constant intensity- comes from transitions between the ground state and many different states which, depending on the magnetic field strength, keep the same symmetry. This argument is also valid for the growing transitions represented by green lines in Fig. 11(a,b). Low-energy allowed transitions are red-shifted by the magnetic field and they decrease and/or increase in intensity; ie., a mixed behaviour can be exhibited depending on the final state of the optical transition. For example, the lowest energy transition in Fig. 11(c) goes from 10 meV to zero when *B* increases from 0 to 30 T and it is forbidden in the range from zero to 5 T (red line); then such transition becomes permitted up to 30 T with increasing intensity. By comparing Fig. 11(b,c) one may conclude that *x*–polarization of the laser field allows a higher energy transition (>40 meV) than *y*–polarized laser radiation.

**Figure 12:**
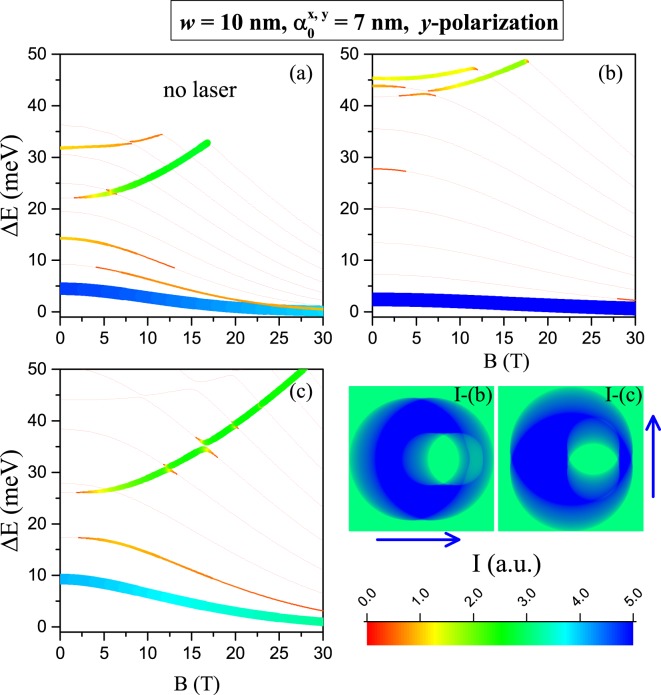
The same as in Fig. 11, but for *y*−polarization of the absorbed light from a probe laser.

Finally, Fig. 12 shows intraband transition energies as in Fig. 11, but with *y*–polarized absorbed light. In comparison with Fig. 11, two conditions may be highlighted: (i) the *y*–polarization favors higher intensities for the lowest intraband transitions; (ii) the highest transitions become not allowed for small applied magnetic field but with magnetic field increase the transitions appear.

## Conclusions

We have performed the investigation of the electronic properties of eccentric GaAs-based two-dimensional quantum rings taking into account the variation of the geometry due to changes in eccentricity, together with the influence of externally applied magnetic and intense nonresonant laser fields. In this context, we present for the first time analytical expressions for the laser-dressed two-dimensional confining potential in this kind of heteroestructures which are valid for all values of the in-plane polarization of the incident laser field. The theoretical study is based on the numerical solution of the effective mass Schrödinger-like partial differential equation with the use of the finite element method.

As a result, a detailed discussion of the properties of the conduction band electron spectrum is given. The changes in the energy level range and distribution, as well as the presence and breaking of state degeneracies are particularly analysed, highlighting the sole or combined influence of the modification in the ring’s geometry and the application of the external electromagnetic fields. Then, with the information on the energy structure at hand, the work extended towards the discussion of the ground-to-excited-states intraband transitions, related to the corresponding optical absorption. This study revealed a broad range of different behaviors, with responses going from few THz to mid infrared. So, the nanostructures considered could become a suitable model for explaining the optoelectronic properties of quantum-ring-based devices^53^.

## Data Availability

All the files with tables, figures, and codes are available. The corresponding author will provide all the files in case they are requested.
